# Influences of Material Selection, Infill Ratio, and Layer Height in the 3D Printing Cavity Process on the Surface Roughness of Printed Patterns and Casted Products in Investment Casting

**DOI:** 10.3390/mi14020395

**Published:** 2023-02-05

**Authors:** Thanh Tan Nguyen, Van Tron Tran, Thi Hong Nga Pham, Van-Thuc Nguyen, Nguyen Chi Thanh, Hong Minh Nguyen Thi, Nguyen Vu Anh Duy, Duy Nguyen Thanh, Van Thanh Tien Nguyen

**Affiliations:** 1 Faculty of Mechanical Engineering, Ho Chi Minh City University of Technology and Education, Ho Chi Minh City 71307, Vietnam; 2 Faculty of Applied Science, Ho Chi Minh City University of Technology and Education, Ho Chi Minh City 71307, Vietnam; 3 School of Mechanical Engineering, Hanoi University of Science and Technology, Ha Noi 113000, Vietnam; 4 Faculty of Engineering and Technology, Nguyen Tat Thanh University, Ho Chi Minh City 71307, Vietnam; 5 Department of Industrial Engineering and Management, National Kaohsiung University of Science and Technology, Kaohsiung 80778, Taiwan; 6 Faculty of Mechanical Technology, Industrial University of Ho Chi Minh City, Nguyen Van Bao Street, Ward 4, Go Vap District, Ho Chi Minh City 70000, Vietnam

**Keywords:** material selection, 3D printing, surface quality, casting products, infill ratio, mechanical properties, investment casting

## Abstract

As 3D-printed (3DP) patterns are solid and durable, they can be used to create thin wall castings, which is complicated with wax patterns because of the wax’s fragility and high shrinkage ratio. According to this study’s experiment results, polylactic acid (PLA), polyvinyl butyral (PVB), and castable wax (CW) are suitable materials for preparing investment casting (IC) cavities. The results indicate that the casting product with the highest-quality surface is obtained using a cavity prepared using a CW-printed pattern. PLA- and PVB-printed patterns provide a good surface finish for casted products. In addition, the roughness of both the printed and casted surfaces increases as the printing layer height increases. The roughness of the casted surface varies from 2.25 μm to 29.17 μm. This investigation also considers the correlation between the infill ratio and mechanical properties of PLA-printed patterns. An increase in the infill ratios from 0% to 100% leads to a significant increase in the tensile properties of the PLA-printed pattern. The obtained results can be practically used.

## 1. Introduction

Investment Casting (IC) is one of the most common casting and mass production methods for creating workpieces since it can produce parts with a high-quality surface [1,2]. IC is a metal casting technique to create complex components such as thin walls, undercut contours, and inaccessible spaces, which are difficult or impossible to manufacture using other fabrication methods [3]. Usually, the IC process consists of several steps [4], which include the creation of wax models using a wax injection machine and assembling them to obtain a complex wax core. Then, the core must be repeatedly dipped into a slurry of refractory materials, such as very fine silica and binders, to develop an outer shell. Afterward, the wax core is removed from the shell at a high temperature (900 °C–1000 °C) to obtain the cavities used for casting molten metals. Finally, the casted metallic part is separated from the shell and cleaned to get the desired workpiece. In the conventional IC, wax with a low melting point is commonly used to construct the core patterns since these sacrificial patterns are entirely removed during autoclaving without ash and damage to the outer shell [5]. However, due to its high brittleness and shrinkage ratio, it is difficult to obtain the complex geometry of the wax core via the assembling process, and the wax patterns will also probably be damaged during the process [6]. This concern is the main issue of using wax in IC. Furthermore, the wax cores are usually prepared using a metal mold, which is timely and costly to machine [4]. Thus, alternative materials that can overcome the wax’s limitations are necessary.

Plastics have good strength, flexibility, and toughness compared to wax; therefore, they can be used to produce thin walls and complex structures. Moreover, the use of plastics also allows finishing operations to improve the surface quality of the designs [7,8]. Recently, many types of 3D printing technology for constructing plastic materials, such as stereolithography (SLA), selective laser sintering (SLS), fused deposition molding (FDM), selective laser melting (SLM), laminated object manufacturing (LOM), digital beam melting (EBM), and digital light processing (DLP) have been rapidly developed [5]. Among them, FDM and SLA are the most common technologies used to print thermoplastics, thermosetting plastics, and composite materials, such as PLA (a biodegradable thermoplastic polyester derived from natural resources [5,9,10,11]), PVB (formed from the reaction of an aldehyde and alcohol [8,12,13]), and CW (a liquid wax [14,15,16]), with complex geometries and a wide range of designs quickly and efficiently [5]. From the above advantages, 3DP and plastics show great potential for applications in IC, which can help to enable a simple production process and provide design flexibility for complex features.

Recently, many efforts have been made to explore the potential application of 3D-printed plastic patterns in IC. The studies investigated printed materials and processing parameters for 3DP to obtain the desired geometries. For instance, to improve the mechanical properties of the printed patterns, acrylonitrile butadiene styrene (ABS) and PC were used as the base and reinforced materials, respectively [4,5,6,10,17]. The tensile properties of PLA structures printed with different infills were investigated [9,17,18,19]. Moreover, to reduce the roughness surface and shell cracking during burnout, the ABS-printed pattern was coated with a thin layer of wax [17]. In other reports [4,19,20,21,22], the effects of printing parameters such as nozzle temperature, nozzle diameter, infill ratio, printing speed, type and many support structures, printing angles, and layer height were studied. In particular, the effect of parameters, including the post-curing process, layer thickness, and water storage on the mechanical properties and degree of conversion of 3D-printed splint material was examined by Perea-Lowery and co-workers [22]. The variation of surface roughness of the printed patterns, along with the interpretation of the printing orientation angle, was also determined in the recent study [19].

**Figure 1:**
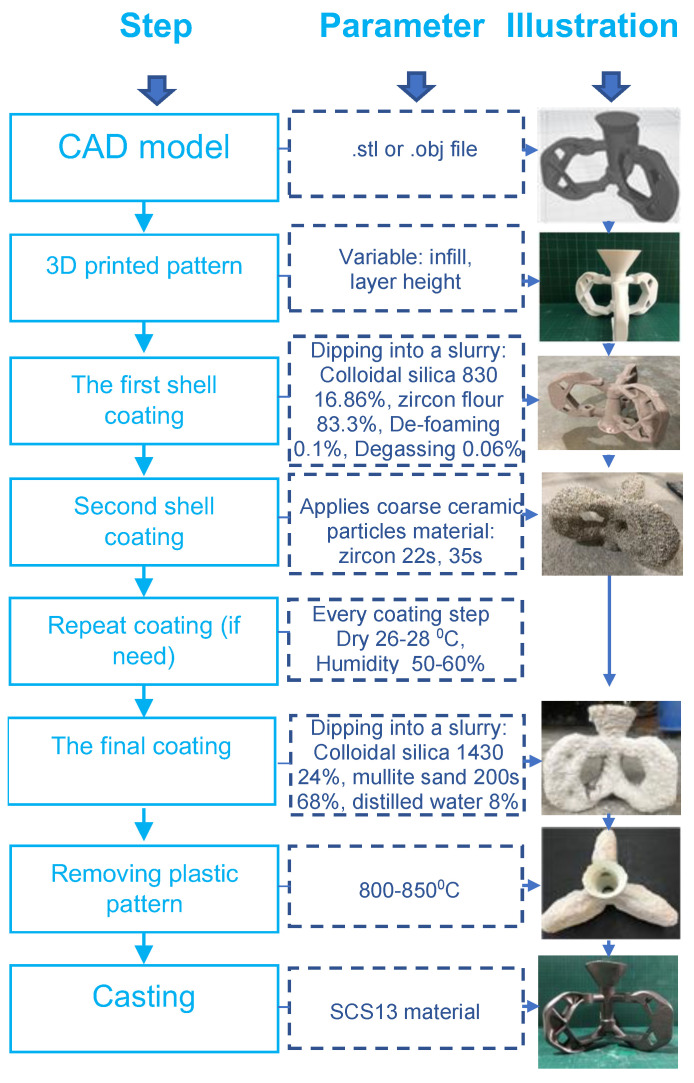
The process for using the plastic–printed pattern in IC.

Although many types of research related to the investigation of printing parameters and mechanical properties of 3D-printed parts in IC have been conducted, it is hard to find reports in the literature about the relations between printing parameters, such as the infill ratio and layer height, the mechanical properties/surface roughness of the printed patterns, or the correlation of the surface roughness between the printed and casted models in IC. To fulfill the technical gaps, in this report, we demonstrated the applications in IC of 3D-printed patterns fabricated using PLA, PVB, and CW, which are widely used in recent related reports that employed the FDM and SLA techniques (see Figure 1). The effects of infill and layer height on the mechanical properties and surface roughness of printed patterns were determined, respectively. Moreover, the relation of surface roughness between the printed and casted parts was also evaluated.

## 2. Materials and Methods

### 2.1. Materials

The PVB and PLA filaments (diameter of 1.75 mm) and liquid CW Resin are supported by 3D Smart Solutions Company, Vietnam. SCS13 steel (JIS G5121 standard) is used as a casting material.

### 2.2. Preparations

**Figure 2:**
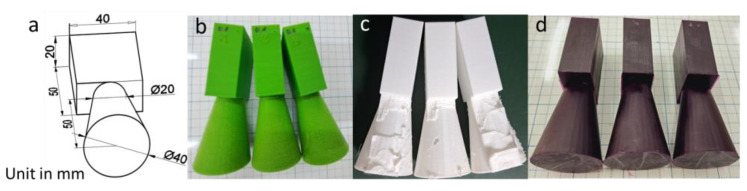
(**a**) Design of the model for the surface roughness test and IC casting. (**b**–**d**) PLA-, PVB-, and CW-printed parts, respectively.

**Figure 3:**
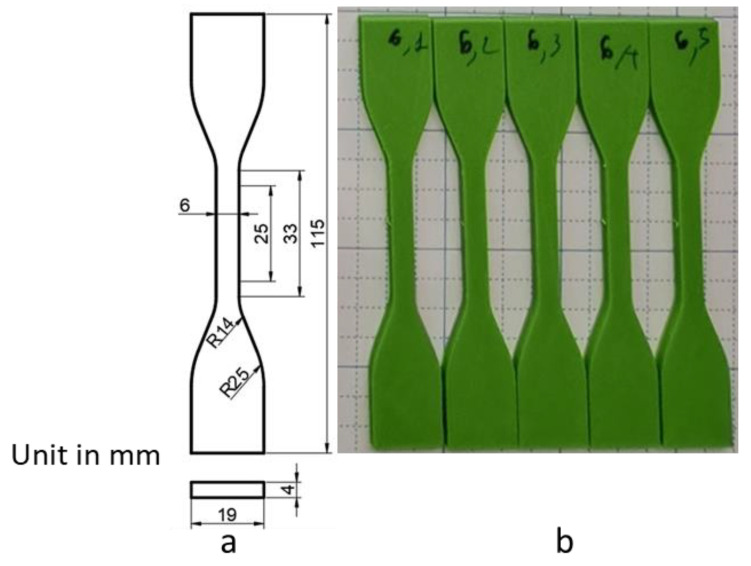
(**a**) Design of the sample for tensile test. (**b**) 3D-printed model using PLA.

- Preparation of 3D–printed patterns. First, the patterns were designed using the 3D AutoCAD software (Autodesk, San Francisco, CA, USA) (Figure 2a). Then, the designs were exported into stereo lithography format (STL) files. Next, they were printed by 3D printers. To prepare the PVB and PLA patterns used for surface roughness tests and IC casting, an FDM printer (supplied by Nam Liem Trading Service Co., Ltd., Ho Chi Minh City, Vietnam) was used with the following parameters: a bed temperature of 60 °C, a nozzle temperature of 210 °C, a printing speed of 60 mm/s, a wall thickness of 0.8 mm, a layer height from 0.06 to 0.6 mm, a fill density of 0%, a grid infill pattern, a non-support material, and a skirt–type build plate (Figure 2b,c). An SLA printer (Formlabs, Somerville, MA, USA) was employed to fabricate the CW pattern with setting layer thicknesses of 0.025 mm and 0.05 mm. Other parameters were used as defaults, which produced the solid pattern (Figure 2d). For the tensile tests, the sample was designed based on ASTD D638 (Figure 3a) and printed by the FDM printer using PLA with the parameters mentioned above, a fixed layer height of 0.06 mm, and variations of infill from 0 to 100% (Figure 3b).
- Preparation of shell molds for IC casting. The printed patterns were repeatedly coated (approximately 4 times) with ceramic as per the sequential process: dipped in a ceramic slurry consisting of 16.86 wt% of colloidal silica 830, 83.3 wt% of zircon flour, 0.1 wt% of de–foaming, and 0.06 wt% of degassing; then covered with ceramic particles (zircon 22 s and zircon 35 s); subsequently dried at 25 °C and humidity of 70%. After that, the plastic patterns were removed from the outer shell in a chamber furnace at 900 °C to obtain the mold cavities. Finally, the molten metal was cast into the cavities (Figure 1).

### 2.3. Methods

- Roughness surface measurements. To evaluate the surface quality of both the printed (PLA, PVB, and CW) and casted parts, the surface roughness (Ra) was measured using a Mitutoyo SJ–201 roughness tester (Mitutoyo, Kawasaki, Kanagawa, Japan) with a cutoff length of 7.5 mm (Figure S1a).
- Tensile test. A tensile test Shimazu 20KN machine (Shimazu Nakagyo, Kyoto, Japan) was used for the tensile test. The sample was clamped on the machine with a gauge length of 50 mm. The upper clamp moved upward at 5 mm/min speed until the sample broke (Figure S1b).

## 3. Results and Discussion

This study used PLA, PVB, and CW to print the cores required to prepare the IC mold cavities. The metallic casting abilities of the cavities were compared, and the effect of the layer height on the roughness of the printed and casted samples was examined. Moreover, the correlation between infill printing and the mechanical properties of PLA–printed models was also investigated.

### 3.1. Evaluation of PLA–, PVB–, and CW–Printed Patterns for Fabricating Metallic Casting Mold Cavities

**Figure 4:**
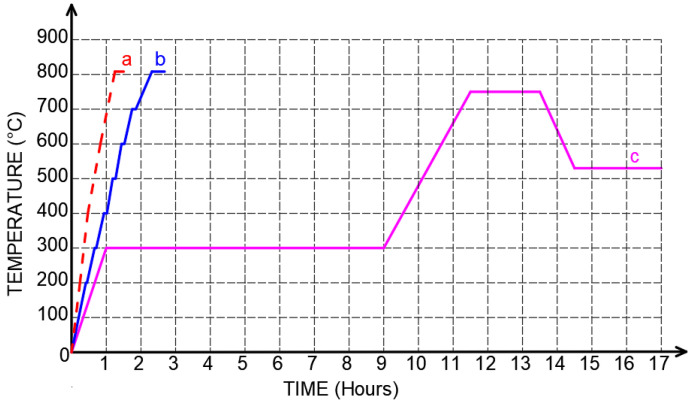
(a) Fast, (b) slow, and (c) multi–step heating rate chart.

After the ceramic outer shells with a thickness of approximately 6 mm were obtained, the plastic–printed patterns were removed by heat in a chamber furnace. To remove the PLA and PVB patterns, the outer shells and the designs underwent two heating processes, including fast and slow heating rates [23,24] (Figure 4a,b). To burn CW, the outer shell and CW pattern were heated using three processes: fast, slow, and multi–step heating rates, followed by the guidelines of the CW producer (Figure 4). In the fast–heating rate process, a temperature of 800 °C was reached after continuously heating for approximately 1.25 h. In contrast, in the slow–heating rate process, after the temperature of 200 °C was obtained, the further increasing temperature was reached step–wise up to 800 °C, in which the temperature was held for 5 min for each increase of 100 °C. The multi–step heating rate (Figure 4c), 300 °C, was reached after 1–h heating. Then this temperature level was held for eight hours. After that, the temperature increased to 750 °C with a heating rate of 4.5 °C/min. Subsequently, a temperature of 750 °C was held for two hours. Next, the temperature was decreased to 512 °C for 1 h. This desired temperature was maintained for 2.5 h.

**Figure 5:**
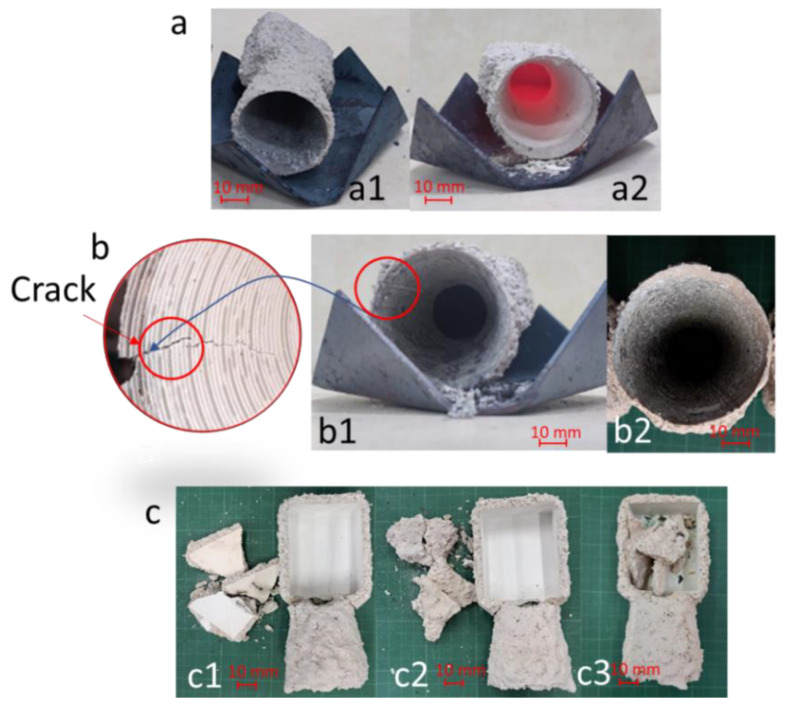
The outer shells were obtained using (**a**) PLA-, (**b**) PVB-, and (**c**) CW-printed patterns. The number (**1**), (**2**), and (**3**) in the figure indicated the fast, slow, and multi–step heating processes, respectively.

The results indicated that after completely removing the PLA and PVB patterns from the ceramic outer shells with both the fast and slow heating processes, in which a negligible remainder of the plastics was observed, the exterior cavities prepared using PLA patterns were obtained without any cracks observed (Figure 5a). In contrast, regarding the cavity fabricated using the PVB patterns, a crack in the outer shell was found after employing the fast–heating rate. However, for the slow–heating rate, the cavity without any cracks was obtained (Figure 5b). By contrast, all of the cavities prepared using CW patterns started cracking and breaking at approximately 200 °C during heating with these processes (Figure S2 and Figure 5c). This result was probably due to the high thermal expansion ratio of the solid CW–printed pattern, which caused a significant expansion of the printed pattern during heating [8]. The above results implied that using the PLA pattern in IC reduces the heating time required to remove the pattern, which can help increase IC productivity. For the PVB pattern, a slow heating rate is necessary to enhance the quality of the obtained cavity for metallic casting. For the CW pattern, further studies are needed to explore its potential application in IC.

**Figure 6:**
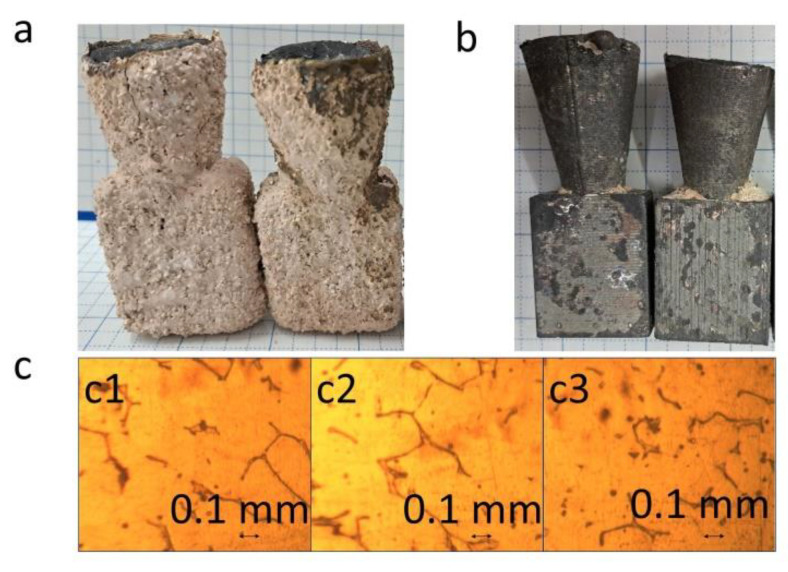
(**a**) Poured metal. (**b**) Casted part. (**c**) Microstructure of casting sample ((**c1**–**c3**) obtained from the wax, PLA, and PVB patterns, respectively).

For the metallic casting, the obtained mold cavities were heated at 950 °C for 50 min. Then, the molten SCS13 steel (at 1680 °C) was poured into the cavities. After that, the molten steel was left to solidify and cool in the air for 2 h (Figure 6a). Finally, the outer cavities were removed to obtain the casted products (Figure 6b), which were used for further characterization. The microstructure of the casted parts was observed using a microscope. The microstructure of the casted parts obtained using plastic patterns was found to be similar to that of the part prepared using wax in conventional IC (Figure 6c). Similar microstructures were obtained since the parts were cast using the molten SCS13 steel designed in the same crucible, and the solidification process was carried out under the same conditions.

**Figure 7:**
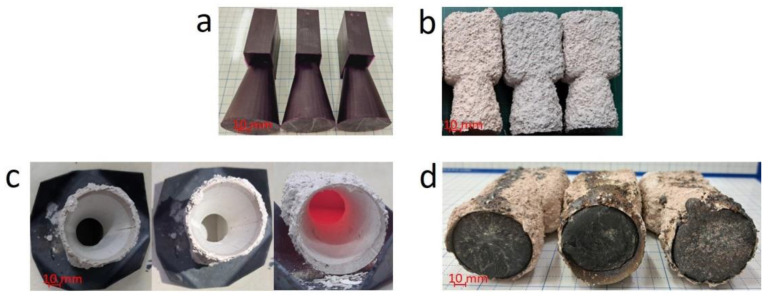
(**a**) 3D-printed part using the hollow design with a wall thickness of 0.8 mm. (**b**) Coated shell. (**c**) Shell mold. (**d**) Casted parts.

It was found that the outer shell prepared using the solid CW patterns cracked and broke during heating (Figure S2 and Figure 5c). The main reason for this issue was due to the high thermal expansion ratio of the CW substance, which caused a significant increase in the volume of solid patterns by heat. Thus, to eliminate this issue, the hollow geometry with a wall thickness of 0.8 mm was designed for a CW–printed pattern, equivalent to 0% infill. With this design, the volume expansion of the pattern was reduced during heating. Therefore, the outer shell without cracks and ash was obtained (Figure 7) [16]. Moreover, the CW pattern printed with the SLA technique exhibited the best quality surface with a Ra of 0.2 μm.

### 3.2. Effect of Layer Height on the Roughness of 3D Printed and Casted Parts

**Figure 8:**
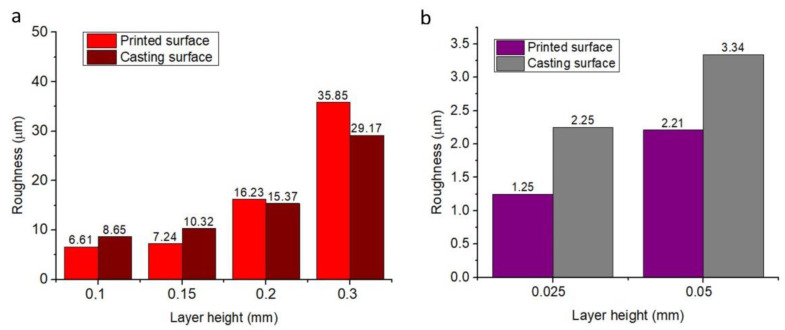
Influence of layer thickness on the surface roughness parameter Ra in the 3D-printed and casted parts. (**a**) Using the PVB pattern. (**b**) Using the CW pattern.

In many recent reports, the roughness parameter, Ra (arithmetic average height), has been widely used to evaluate the surface roughness of both printed and casted products in IC [8,19,20,21]. Thus, in this study, the roughness Ra of the printed and casted models’ surface was also measured using a Mitutoyo SJ–201 roughness tester (Figure S1a). Morover, the correlation of surface roughness of the models was indicated. For PLA, in the beginning, the surface roughness (Ra) of the printed surface moderately increased from 6.1 μm to 15.4 μm with an increase in the printing layer height from 0.06 mm to 0.2 mm. Then, this value dramatically increased up to 42.8 μm and further increased the layer height to 0.6 mm (Table 1 and Figure S3). The increase in the surface roughness of the PLA–printed pattern led to the rise in the surface roughness of the casted part from 6.4 μm to 26.5 μm (Table 1 and Figure S3). Moreover, the results also indicated that in a range of layer height from 0.06 mm to 0.2 mm, the roughness of the printed surface was lower than that of the corresponding casted surface. In contrast, when the layer height increased from 0.3 mm to 0.6 mm, the surface roughness of the former was higher than that of the latter (Table 1 and Figure S3). This phenomenon was probably due to the high surface tension of the molten metal, which prevented it from fulfilling the micro slots in the printed surface during casting [13,25,26]. For the PVB pattern, the correlation of surface roughness between the printed and casted models was similar to that of the PLA–printed pattern. The surface roughness of both the printed and casted parts increased from 6.61 μm to 35.85 μm and 8.65 μm to 29.17 μm, respectively, together with growing layer height from 0.1 mm to 0.3 mm (Figure 8a). Furthermore, at the beginning of growing layer height from 0.1 mm to 0.2 mm, the surface roughness of the printed surface was lower than that of the casted surface, whereas with the layer height of 0.3 mm, the opposite results were obtained (Figure 8a). For the CW patterns, with an increase in the layer height from 0.025 mm to 0.05 mm, the surface roughness of both the printed and casted parts increased, and the casted surface was rougher than that of the printed surface (Figure 8b).

### 3.3. Effect of Infill Printing on Mechanical Properties of PLA Material

**Figure 9:**
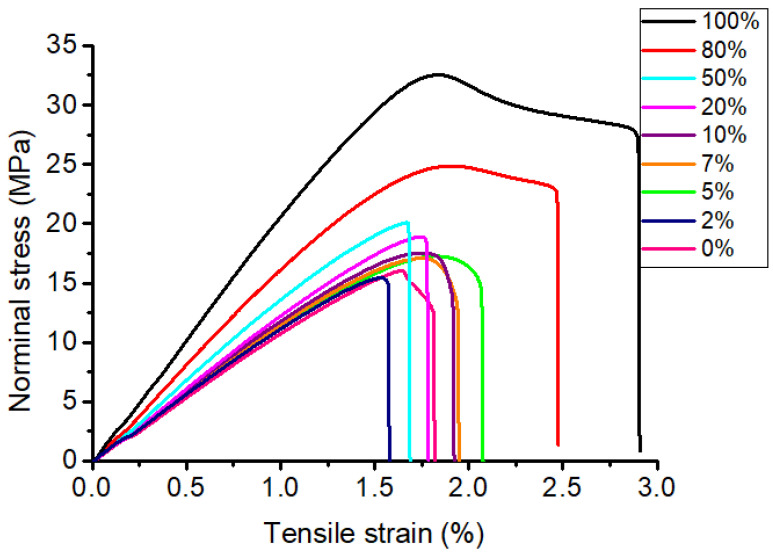
The tensile stress–strain curves of the PLA with the variations of infill ratio.

Investigating the effect of printing infill on the mechanical properties of the printed pattern, the tensile test specimens were printed following the ASTM D638 standard and using PLA plastic (Figure 3). During printing, the layer height was set at 0.2 mm, and the infill was varied from 0 to 100%. The variations of the infill density selected in this study were based on recent research [27,28]. With an increase in the infill ratio from 0 to 100%, the printing time and weight of the printed specimen gradually increased (Table S1 and Figure S4). To evaluate the mechanical properties of the printed specimens, tensile tests were carried out (Figure S1b). The results indicated that the tensile strength of printed structures significantly depended on the infill ratio. In particular, the specimen with 100% infill exhibited a tensile strength of 32 MPa (Figure 9 and Figure S4, Table 2). In comparison, the tensile strength drastically decreased by 21.5%, 37.06%, 40.56%, 45.34%, and 49.69%, corresponding to the specimens with infill ratios of 80%, 50%, 20%, 10%, and 0%, respectively (Figure 9 and Figure S4, Table 2). This concern was ascribed to a decrease in the material density of the printed specimens, along with a reduction in the infill ratio. However, the tensile strength of the PLA specimens was much higher than that of the wax substance [29,30,31]. The tensile strain of the specimens slightly decreased at the beginning from 100% to 50% of the infill ratio and then almost maintained (Figure 9 and Figure S4, Table 2).

## 4. Conclusions

This study systematically evaluated the potential application of PLA–, PVB–, and CW–printed patterns in IC. To prepare the casting cavity, the PLA–printed pattern could be quickly removed with the fast–heating rate process, while the slow–heating rate process was rational to burn out the PVB pattern. For the CW pattern, the hollow printed structure was needed to prevent the outer shell from cracking and breaking during heating. The correlations between the printing layer height and surface roughness of the printed surface and the surface roughness of the printed and casted surfaces were investigated. An increase in the printing layer height led to a rise in the surface roughness of both the printed and casted surfaces. Moreover, at a low layer thickness, the surface roughness of the printed part was lower than that of the cast one. At the high layer thickness, the opposite results were displayed. The effect of the infill ratio on the mechanical properties of the PLA printed specimen was also determined. A decrease in the infill resulted in a reduction of tensile strength, while the tensile strain slightly decreased at the beginning and then almost remained constant. Our research results provided more insight into the potential application of these plastics in IC and could be employed in practical casting.

## Figures and Tables

**Table 1:** The roughness and optical microscopic images (OMI) of the PLA–printed surface and casted surface vary with infill density and layer height.

Layer Height (mm)	3D Printed Sample		Casted Sample	
	Roughness (μm)	OMI	Roughness (μm)	OMI
0.06	6.1	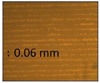	6.4	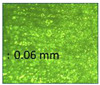
0.1	8	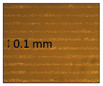	10	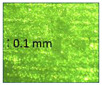
0.15	10.6	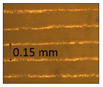	11.5	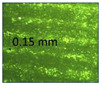
0.2	15.4	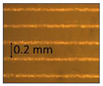	15.6	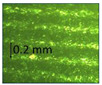
0.3	24.7	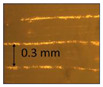	21.7	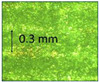
0.4	33.6	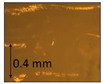	21.5	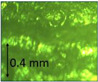
0.6	42.8	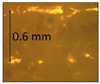	26.5	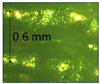

**Table 2:** Mechanical properties of PLA material with a variation of infill density.

Items	Infill (%)								
	0	2	5	7	10	20	50	80	100
Tensile strength (MPa)	16.72	16.10	17.49	17.18	17.37	19.02	20.14	25.12	32
Tensile strain (%)	1.92	1.74	2.02	2.04	1.99	1.98	1.73	2.57	2.54

## Data Availability

The data used to support the findings of this study are available from the corresponding author upon request.

## Supplementary Materials

The following supporting information can be downloaded at: https://www.mdpi.com/article/10.3390/mi14020395/s1, Figure S1: (a) Roughness test and (b) tensile test; Figure S2: The outer shells prepared using CW patterns cracked and broke at 200 °C during (a) slow–heating rate, (b) fast–heating rate, and (c) multi–step heating rate processes; Figure S3: Influence of layer thickness on the surface roughness in 3D printed part using PLA and casted product; Figure S4: Printing time, weight, and tensile properties of the printed patterns using PLA with the variation of infill density; Table S1: Printing time and weight of PLA printed pattern with the variation of infill density.
