# Supplementary material for: Influences of Material Selection, Infill Ratio, and Layer Height in the 3D Printing Cavity Process on the Surface Roughness of Printed Patterns and Casted Products in Investment Casting
Source: Micromachines (Basel). 2023 Feb 5;14(2):395. doi: 10.3390/mi14020395 (PMC9965826; doi:10.3390/mi14020395)
Supplement: Supplementary file 1 [file micromachines-14-00395-s001.zip › micromachines-2098198-supplementary.pdf]

Supporting Information

Influences of Material Selection, Infill Ratio, and Layer Height in the 3D Printing Cavity Process on the Surface Roughness of Printed Patterns and Casted Products in Investment Casting

Thanh Tan Nguyen [1], Van Tron Tran [1,*], Thi Hong Nga Pham [1], Van-Thuc Nguyen [1], Nguyen Chi Thanh [2], Hong Minh Nguyen Thi [3], Nguyen Vu Anh Duy [4], Duy Nguyen Thanh [1] and Van Thanh Tien Nguyen [5,6,*]

[1] Faculty of Mechanical Engineering, Ho Chi Minh City University of Technology and Education, Ho Chi Minh City 71307, Vietnam
[2] Faculty of Applied Science, Ho Chi Minh City University of Technology and Education, Ho Chi Minh City 71307, Vietnam
[3] School of Mechanical Engineering, Hanoi University of Science and Technology, Ha Noi 113000, Vietnam
[4] Faculty of Engineering and Technology, Nguyen Tat Thanh University, Ho Chi Minh City 71307, Vietnam
[5] Department of Industrial Engineering and Management, National Kaohsiung University of Science and Technology, Kaohsiung 80778, Taiwan
[6] Faculty of Mechanical Technology, Industrial University of Ho Chi Minh City; Nguyen Van Bao Street, Ward 4, Go Vap District, Ho Chi Minh City 70000, Vietnam
* Correspondence: trontv@hcmute.edu.vn (V.T.T.); thanhtienck@ieee.org (V.T.T.N.)

Supporting figures

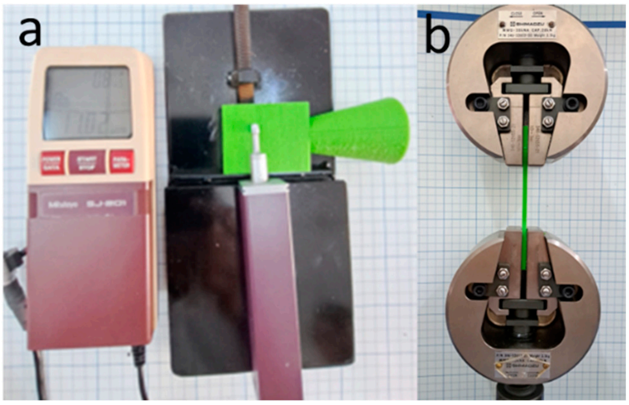

Figure S1. (a) Roughness test and (b) tensile test.

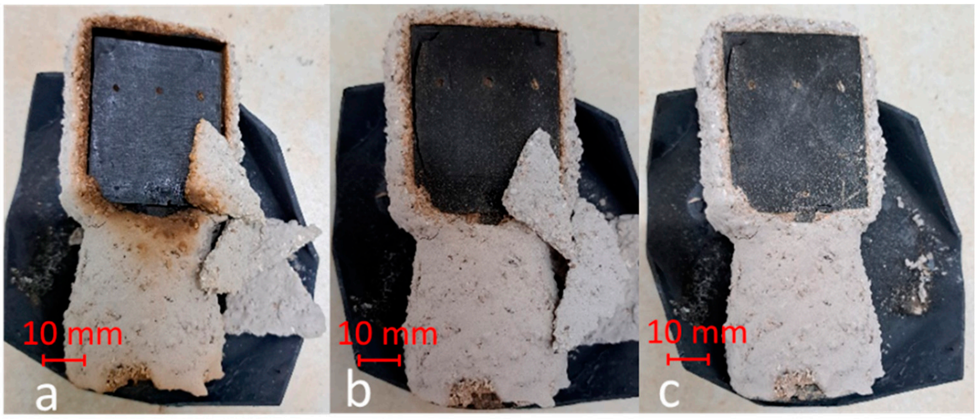

Figure S2. The outer shells prepared using CW patterns cracked and broke at 200 °C during (a) slow-heating rate, (b) fast-heating rate, and (c) multi-step heating rate processes.

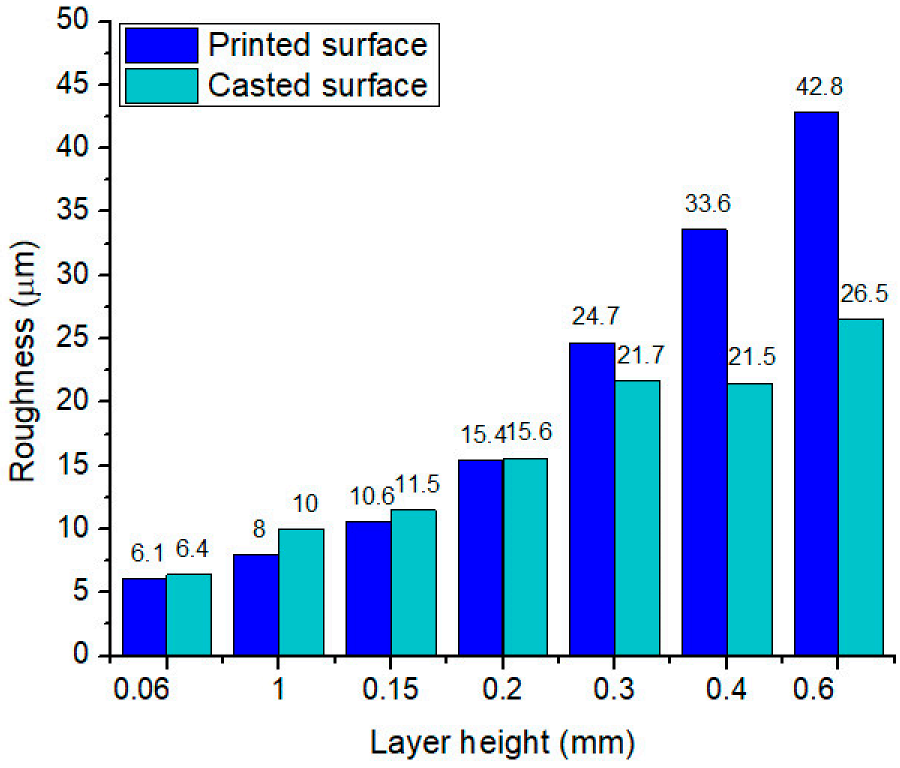

Figure S3. Influence of layer thickness on the surface roughness in 3D printed part using PLA and casted product.

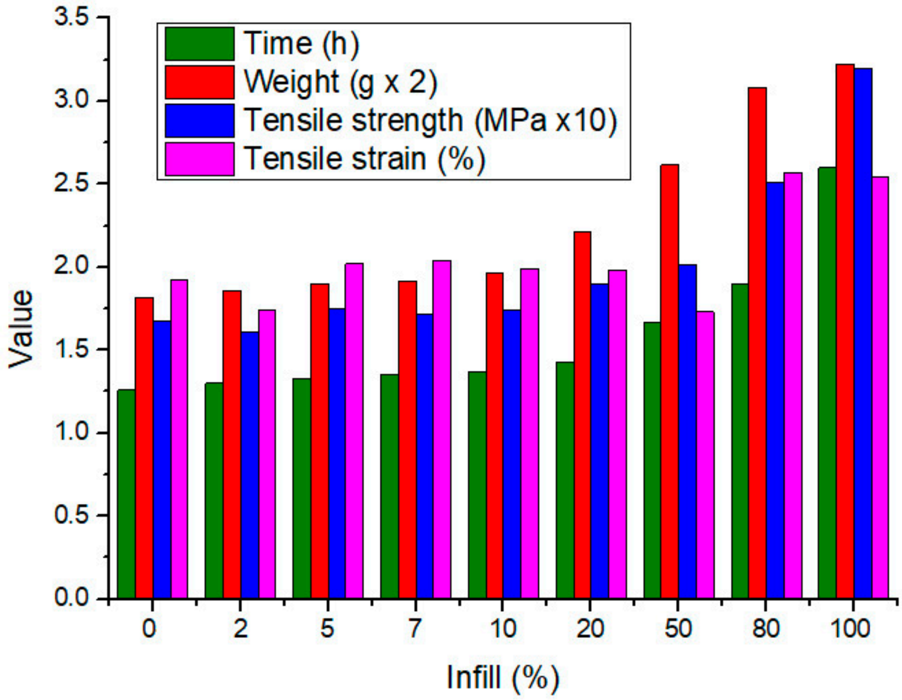

Figure S4. Printing time, weight, and tensile properties of the printed patterns using PLA with the variation of infill density.

Supporting table

Table S1. Printing time and weight of PLA printed pattern with the variation of infill density.

**Items**	**Infill (%)**								
	**0**	**2**	**5**	**7**	**10**	**20**	**50**	**80**	**100**
Time (h)	1.27	1.30	1.33	1.35	1.37	1.43	1.67	1.9	2.6
Weight (g)	3.63	3.72	3.80	3.83	3.92	4.25	5.23	6.17	6.45
